# Behaviour of anadromous brown trout (*Salmo trutta*) in a hydropower regulated freshwater system

**DOI:** 10.1186/s40462-023-00429-7

**Published:** 2023-10-14

**Authors:** Lotte S. Dahlmo, Gaute Velle, Cecilie I. Nilsen, Ulrich Pulg, Robert J. Lennox, Knut W. Vollset

**Affiliations:** 1 LFI Laboratory for Freshwater Ecology and Inland Fisheries, NORCE Norwegian Research Centre, Nygårdsgaten 112, 5008 Bergen, Norway; 2 Department of Biological Sciences, University of Bergen, Thormøhlens Gate 53A, 5008 Bergen, Norway; 3 NINA Norwegian Institute for Nature Research, Høgskoleringen 9, 7034 Trondheim, Norway

**Keywords:** Biologging, Anadromous brown trout, Hydropower, Lake ecology, Acceleration

## Abstract

Many Norwegian rivers and lakes are regulated for hydropower, which affects freshwater ecosystems and anadromous fish species, such as sea trout (*Salmo trutta*). Lakes are an important feature of many anadromous river systems. However, there is limited knowledge on the importance of lakes as habitat for sea trout and how hydropower affects the behaviour of sea trout in lakes. To investigate this, we conducted an acoustic telemetry study. A total of 31 adult sea trout (532 ± 93 mm total length) were captured by angling in river Aurlandselva, Norway, and tagged between July 20 and August 12, 2021. The tags were instrumented with accelerometer, temperature, and depth sensors, which provided information on the sea trout’s presence and behaviour in lake Vassbygdevatnet. Our results indicate that there was a large prevalence of sea trout in the lake during the spawning migration, and that the sea trout were less active in the lake compared to the riverine habitats. An increase in activity of sea trout in the lake during autumn might indicate that sea trout spawn in the lake. However, the discharge from the high-head storage plant into the lake did not affect the depth use or activity of sea trout in the lake. Furthermore, the large prevalence of spawners in the lake during autumn will likely cause an underestimation of the size of the sea trout population in rivers with lakes during annual stock assessment. In conclusion, our results could not find evidence of a large impact of the discharge on the behaviour of sea trout in the lake.

**Supplementary Information:**

The online version contains supplementary material available at 10.1186/s40462-023-00429-7.

## Introduction

Freshwaters comprise only a small fraction of the Earth, yet freshwater habitats are disproportionately threatened by overexploitation, pollution, and regulation [22, 26, 65, 91]. Salmonids and other species that rely on freshwater are therefore vulnerable [43], and changes to rivers and lakes can impact resident and migratory fish populations [11, 58]. Hydropower regulations can cause changes to the natural water flow, such as the timing, magnitude, and variability of the water flow [59, 77]. Hydrological changes affect both the biotic and abiotic variables upstream and downstream of modified areas by altering the movement of sediments and organic resources, availability of habitat types, shelters, and forage opportunities, and the distribution, abundance, and richness of species [59, 60, 82]. The effects of regulation and modifications of rivers on freshwater fish are frequently studied (e.g., [11, 69]) and restoration interventions (e.g., fishways, barrier removal, gravel augmentation) are increasingly implemented to improve habitats, such as the connectivity or quality (e.g., [44, 61, 63, 66]). In contrast, there is a lack of studies on how hydropower impacts lake habitat for anadromous species [46].

Norwegian rivers and lakes are highly exploited to generate hydropower due to a topography with an abundance of freshwater systems across different altitudes, steep mountains, and high annual precipitation [1]. In contrast to run-of–river hydropower plants that produce energy by implementing physical barriers, such as dams and weirs in rivers [3, 8], the topography of Norway allows for high-head storage plants [1]. Storage plants exploit the potential energy of water from reservoirs and often discharge into lakes, which are important habitats for anadromous brown trout (hereafter referred to as sea trout, *Salmo trutta*) and Atlantic salmon (*Salmo salar*, [46]). The intake of high-head storage plants is often in the deeper part of reservoirs, which results in the transfer of hypolimnetic water through turbines and into a fjord, river, or a reservoir, such as a natural lake or artificial reservoir [31]. The hypolimnetic water (~ 4℃) transported by the storage plant therefore supplies relatively cold water during summer and warm water during winter [31, 67]. More than 30% of Norwegian rivers run through lakes, many of which are highly exploited to generate hydroelectricity [1] and may also involve migration barriers, such as weirs and dams.

Animal choice of habitat depends on a trade-off between their energy budget (i.e., growth) and mortality rate [85]. Alteration of habitats can affect behaviour and accelerate energy depletion of animals [36], for instance through increasing movement and activity. A logical question is therefore whether high-head storage plants increase the activity level of sea trout in lakes and alter their habitat choice. This study aims to provide insight into the lake use and activity of sea trout by measuring their movement in the three spatial axes. By using acoustic transmitters (i.e., tags) equipped with acceleration and depth sensors, we investigated whether adult sea trout in a watercourse including a lake used the lake before spawning and whether their behaviour was affected by discharge from the high-head storage plant. Specifically, we hypothesised that: 1) the lake is used by sea trout before spawning, and that 2) the activity (acceleration) of sea trout is higher in the rivers than in the lake, and 3) the high-head storage plant discharge alters the behaviour of sea trout during the spawning migration.

## Methods

### Study site

**Fig. 1:**
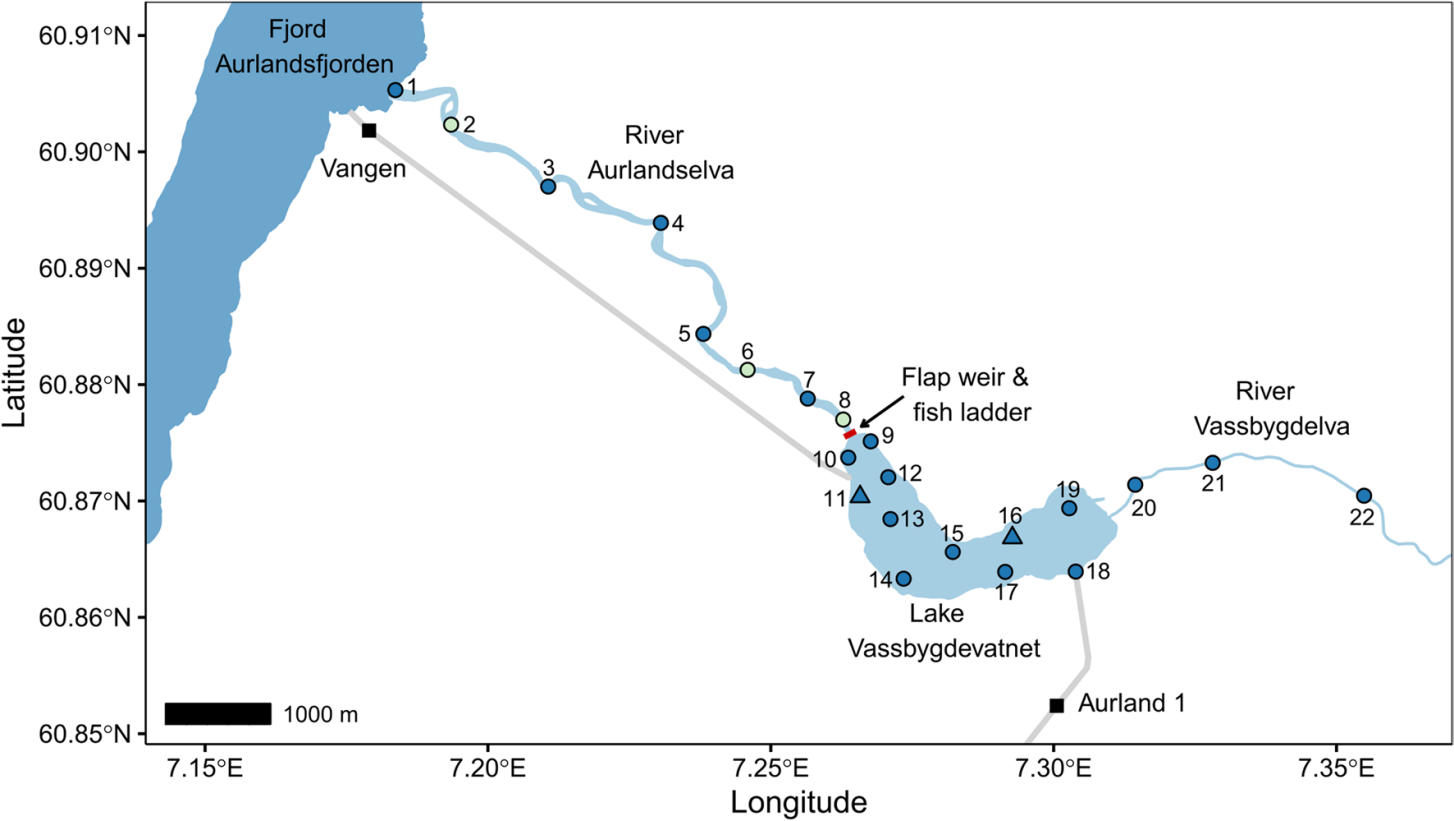
Map of Aurland watercourse with the location of receivers (circles, triangles) deployed prior (blue) and post (green) tagging, the ‘Aurland 1’ high-head storage plant, ‘Vangen’ storage plant, and the flap weir and fish ladder at the outlet of Lake Vassbygdevatnet (red line). Two synchronization transmitters were placed with two of the receivers in the lake (triangles). Receivers are numbered between 1–22 for identification

The study was conducted in the Aurland watercourse in Vestland county, Norway (Fig. 1). The upstream river Vassbygdelva runs from the mountains and constitute the main river inflow into lake Vassbygdevatnet. Anadromous fish can migrate up nearly 5 km of the lowest reaches of river Vassbygdelva until steep areas act as natural barriers hindering further migration (see Fig. 1. in [62, 80]). Vassbygdevatnet has a length of 3.3 km, covers an area of 1.9 km^2^, and has an average depth of 42 m and maximum depth of 65 m. The river Aurlandselva, runs 6.7 km downstream from the lake before it ends in the fjord Aurlandsfjorden, an arm in the Sognefjord about 170 km from the open ocean. In Aurland, sea trout can inhabit approximately 15 km of the watercourse [80]. Following the river regulation from 1969, both Atlantic salmon and trout populations exhibited a dramatic decline by the late 1980s [80]. Today, the sea trout population dominates and has large recreational value to anglers and great socio-economic importance to the local community, while the salmon population is still significantly reduced and has been protected since 1989 [37, 63].

#### Hydropower plants

The construction of the hydropower system in Aurland began in 1969 and lasted until 1989 [80]. Today, the hydropower system consists of five power plants, which together with 14 reservoirs and several tunnels, regulate the Aurland watercourse [80]. Two of these power plants directly influence the lake Vassbygdevatnet in Aurland (Fig. 1). The ‘Aurland 1’ plant is a high-head storage plant (850 m in head height, 840 MW) with outlet running into the southeastern part of Vassbygdevatnet and is the largest power plant in the watercourse. Aurland 1 constitutes the primary supply of water into the lake by transporting water from the mountain reservoirs. Therefore, the lake surface temperature is impacted, being colder during summer and warmer during the winter, which results in a low thermal stratification of the lake [80]. The ‘Aurland 4’ storage plant (55 m in head height, 38 MW), also known as ‘Vangen’, has its intake in the western part of lake Vassbygdevatnet that leads to a tunnel running down to the power plant by the fjord. The Vangen station operates from September 15 until the end of April, and during this period a flap weir located at the outlet to river Aurlandselva is elevated, thereby regulating the water flow downstream in the river (Fig. 1). While Vangen is operating, Aurlandselva has a mandatory minimum discharge of 3 m^3^/s that is upheld by release of water over the flap weir [94]. The lake functions as a semi-natural reservoir while Vangen is operating. A pool and weir fish ladder along the west side of the flap weir allows for fish migration between the lake and the river (head 1–2 m) when the weir is elevated.

#### Discharge data

Aurland 1 released an average discharge of 20.97 m^3^/s (± 16.95) into the lake during the study period (July 20–November 14, 2021, see Additional file 1: Figure S1), with a minimum discharge of 0 m^3^/s and a maximum discharge of 108.46 m^3^/s. Before the flap weir was elevated, the downstream river Aurlandselva had an average discharge of 27 m^3^/s (± 10.40) and a minimum and maximum discharge of 3.75 and 51.11 m^3^/s, respectively. After the elevation of the flap weir, the average discharge was 4.25 m^3^/s (± 0.54), the minimum discharge was 2.96 m^3^/s, and the maximum discharge was 8.13 m^3^/s in Aurlandselva. Discharge data for the study period were provided by the hydropower company Hafslund ECO.

### Study design

All sea trout were captured, tagged, and released between July 20 and August 12, 2021. Prior to capturing fish, a total of 19 TBR 700 and 700L acoustic receivers (Thelma Biotel AS, Trondheim, Norway) were deployed: three in river Vassbygdelva; five in river Aurlandselva; and eleven in lake Vassbygdevatnet (Fig. 1). Two synchronizing transmitters (“sync tags”) were deployed with two receivers to correct clock drift of the receivers in the lake. Three additional receivers were deployed September 2 in river Aurlandselva after all fish were captured, tagged, and released, to maximize the coverage in the river during the autumn migration and spawning (Fig. 1). Data were downloaded from all 22 receivers on November 15 and 16, 2021.

### Sampling and tagging

A total of 31 sea trout (540 ± 102 mm total length) were captured by recreational anglers in river Aurlandselva. All sea trout were tagged and released in proximity of where they were caught, with a total of nine capture sites located between the confluence of the lake and the river, and the site furthest down (close to the river mouth). Sea trout were kept in keepnets or tubes for a minimum of 30 min after hooking to provide a recovery period. Most sea trout were caught during night and tagged within 6 h the following morning, and a few sea trout were held up to 20 h before being tagged and released. To ensure a tag burden less than 2% of body weight (e.g., Jepsen et al. 2005; [76]), a lower weight limit was converted to a lower length limit of fish by using Fulton’s condition formula [66] with an assumed K value of 1. The minimum total fish length was calculated to 38 cm, and the smallest fish tagged was 41.5 cm. Thus, the maximum tag burden was approximately 1.6% of the fish’s body weight. To avoid selection of sea trout, all captured sea trout above the minimum total length requirement in the present study were assessed suitable for tagging by visual assessment (any visible wounds, marks, or lice) and response to external stimuli were checked. Remarks on visible wounds, marks, or lice were noted, however, none of the sea trout had severe external marks or wounds, or was assessed to be in an unsuitable condition for tagging.

Prior to surgery, each sea trout was anaesthetized with 1.5–2 mL Aqui-S in a container with 50 L water until equilibrium was lost (6–9 min). The fish was placed supine in a tube where fork length (mm) and total length (mm) were measured. A silicone tube with running water containing 50% dose of the anaesthetics was placed in its mouth to maintain anaesthesia and oxygenation during surgery. A 15–18 mm incision was performed with a sterile scalpel approximately 3 cm posterior to the pectoral fins and 1–2 mm from the linea alba. The sterilized LP13-ADT acoustic tag (S64K protocol, 90 s nominal delay, 11.5 g in air, 33.3 mm long, 13 mm wide; Thelma Biotel, Trondheim, Norway) was placed into the abdomen, followed by three interrupted sutures to close the incision. The sensors had a range between 0 and 255, thus any values above maximum were registered as 255. The surgery, including the anaesthetic period, lasted for approximately 16 min. Tagged fish were transferred to keepnets or containers with fresh river water and observed during recovery for about ten to fifteen minutes before being released. Every fish was tagged and released close to its capture site (hereafter referred to as tagging site). Approval of the project was given by the Norwegian Food Safety Authority (FOTS, application nr. 23016), and handling and tagging of sea trout was conducted according to the Norwegian animal welfare regulations.

### Data analysis

All preparation, visualization, and statistical analyses of data were conducted in R-Studio 4.1.2 [64]. Positions were derived for all individuals in the lake based on multilateralization of the detections in the receiver grid. Transmissions of two synchronisation tags (Fig. 1) were used to synchronise the receiver clocks in the lake using Yet another positioning solver (YAPS, [7]) function *getSyncModel* with an eps threshold of 10. A custom wrapper function for the YAPS algorithm was written to fit five model fits to each fish day in the time series and select the model with the best fit. Positions with estimated error > 20 m in both the x and the y dimensions were discarded.

Acoustic telemetry and detection data are prone to false detections [70], which is necessary to account for. False detections were identified and removed with cleaning tools (such as the filter(), mutate(), and case_when() functions) in the *dplyr* package [88]. Data were visualized with the *ggplot2* package [87] and model interpretations were visualized with the *gratia* package [71].

All generalized additive models (GAMs) used in the data analyses were implemented with the *bam()* function from the *mgcv* package [90], which is suitable for larger datasets. Additionally, a gamma distribution with a log link function was used in all the GAM models. The gamma distribution was used because the response variable of the models was continuous and positive [92]. The collinearity between explanatory variables was checked with the *ggpairs()* function from the *GGally* package [68] to exclude variables that were correlated. To test whether the smoothers (term to account for non-linear variation over time) followed the same pattern, the *concurvity()* function from the *mgcv* package was used. The function calculates three measures of concurvity (worst, observed, and estimate), and by using the concurvity values from the most pessimistic measure (worst), values above 0.8 indicates strong presence of concurvity [21] and therefore similar patterns between two smoothers.

The raw dataset was filtered so that only data from the study period (July 20–November 14, 2021) and the unique IDs from the S64K-69 kHz protocol were retained in the dataset. One individual ID (ID 4697) died or lost its tag one month after tagging. For this individual, only detections up until August 26, 2021, were included. One fish (ID 4685) was never detected, giving a final sample size of 30 sea trout. Nine additional detections from three individuals were manually removed following closer inspection of the raw dataset. To account for any additional potential false detections, three filtering codes with different criteria were constructed and any detections that met the criteria were removed. The dataset was first filtered by grouping the dataset by fish ID, then calculating the speed (m/s) and distance (m) from the previous detection. Therefore, the first detection from each unique fish had a distance and speed equal to zero. The three filtering codes were: 1) detections from one of the river receivers where the previous detection was in the lake and the distance calculated was greater than 1000 m; 2) detections from a lake receiver with a previous detection from one of the river receivers and a calculated distance greater than 1000 m; and 3) any detections with a distance larger than 800 m and with a speed greater than 5 m/s. The speed criteria was set to 5 m/s as it is unlikely that salmonids swim faster than 5 m/s over longer distances [23], [56].

#### Hypothesis 1: Habitat use

The time spent in the two habitats (i.e., river or lake) was calculated by assigning each individual to a habitat at any given minute after their respective tagging day until the end of the study period. For undetected time stamps, habitat was interpolated using the previous habitat that an individual was detected in. For the time between the tagging and first detection, the habitat was interpolated using the first habitat an individual was detected in.

In order to test whether the lake is an important habitat for sea trout before spawning, a generalized linear model was built with a poisson distribution by using the *glm()* function in R with the number of trout in the lake as a response variable (*lake*). All individuals were assigned to either river or lake per minute throughout the study. Thus, undetected minutes per individual were interpolated by using the previous habitat a fish was detected in. Day of year (*day*) and the number of sea trout that could have been in the lake (calculated by offset; *total*) were used as explanatory variables. The model was given as:

**Model 1.1**

Lake ~ day + offset(log(total)), family = “Poisson”.

#### Hypothesis 2: Effect of habitat on activity

To investigate the effect of habitat on the activity, acceleration (m/s^2^) was used as proxy for activity as demonstrated by Mulder et al. [52] with Arctic charr (*Salvelinus alpinus*) in a similar environment. This sensor is a tri-axial accelerometer with a range of 0–3.465 m/s^2^ that measures both dynamic and static acceleration. The tags were programmed to measure acceleration in the three axes for 27 s at 12.5 Hz and then calculate a root mean square value summarising the three axes, encoding this value as a number between 0 and 255 (the integer range of the sensor), and transmitting this value to the receiver. The raw acceleration being a value between 0 and 255, this was transformed back to root mean square (RMS) using the following equation: RMS = raw data × 3.456/255.

To test whether the activity of sea trout differs between the rivers and the lake, a GAM model was built. Activity based on accelerometer data (or acceleration (m/s^2^), *accel*) was modelled as the response variable, while *habitat* (lake or river, as factor), day of year (denoted as *day*), and time of day (*time*) were included as explanatory variables. The unique fish ID (*individual*) variable was included as a random effect. There was high correlation between day of year and discharge in the downstream river (-0.835), and high correlation between day of year and temperature (-0.944, temperature measured from temperature sensor in the tags). Temperature and the discharge in the downstream river were therefore not included, to retain the temporal structure of the variance in the models.

A smoother (*s()*) was used for each of the temporal variables (day and time) to account for non-linear variation over time. When the wiggliness of values of a variable differ substantially, it can be useful to include an interaction in the smoother, which informs the model to apply a separate smoother for each level of a factor [57]. The term ‘*by* = *habitat*’ was included in each of the temporal smoothers so that a smoother was fitted to each level of habitat (i.e., lake and river). For the random effect of fish ID, a smoother was used to account for nestedness and repeated measurements of observations, with “re” specifying that the basis for smoothing (bs) is adjusted to the random effect of the variable and k equals to the sample size (k = N = 30). The amount of wiggliness (k) was adjusted to the other smoothers.

Because the dataset was built up by repeated measurements from the same sea trout individuals over time, an autocorrelation term was included to test if the autocorrelation structure improved the model. The autocorrelation term was calculated based on the first model and then included in the second model. Akaike Information Criterion (AIC; [39] was used to compare the fit of the two models. The final models were:

**Model 2.1**

Accel ~ habitat + s(day, by = habitat, k = 40) + s(time, by = habitat, k = 10) + s(individual, bs = "re", k = 30), method = "fREML", family = Gamma(link = "log").

**Model 2.2**

Accel ~ habitat + s(day, by = habitat, k = 40) + s(time, by = habitat, k = 10) + s(individual, bs = "re", k = 30), AR.start = starting_timepoint, rho = rho_value, method = "fREML", family = Gamma(link = "log").

#### Hypothesis 3: Effect of high-head storage plant discharge on behaviour in the lake

To test if the high-head storage plant discharge alters the behaviour of sea trout during the spawning migration, GAM models were built with- and without the discharge as an explanatory variable based on a subset of the data only from the lake. The models were built by the explanatory variables day of year (*day*), time of day (*time*), and a bivariate smoother to account for the spatial interaction between longitude (*longitude*) and latitude (*latitude*) calculated from the YAPS positioning algorithm. The spatial smoother had a k-value of 100 to allow for large spatial variation. A smoother was also used for each of the two temporal variables to account for seasonal- and daily variation in depth use. To account for the random effect of individual sea trout, the fish IDs (*individual*) was included as a factor in a smoother, with k equal to the number of sea trout detected in the lake (k = N = 26). A calculated autocorrelation structure was included in all models. The discharge data from the high-head storage plant Aurland 1 (*AU1*) was included as an additional explanatory variable. Four models were built to investigate the effect of the discharge on the depth use and activity in the lake independently, with average depth (*depth*, model 3.1 and model 3.2) and activity (*activity*, model 3.3 and model 3.4) as response variables in two of the models each. For the two models of activity in the lake (model 3.3 and model 3.4), depth (*depth*) was added as an additional explanatory variable in the two models. An AIC model comparison was implemented to test whether the discharge data from the storage plant improved the model fit. The best fitted models (one for depth and one for activity) were visualised for inspection of the explanatory variables by drawing predictions from the model output on a grid of all possible values in the data series. The four models were:

**Model 3.1**

Depth ~ s(longitude, latitude, k = 100) + s(day, k = 40) + s(time, k = 4) + s(individual, bs = "re", k = 26), AR.start = starting_timepoint, rho = rho_value, method = "fREML", family = Gamma(link = "log").

**Model 3.2**

Depth ~ s(AU1, k = 4) + s(longitude, latitude, k = 100) + s(day, k = 40) + s(time, k = 4) + s(individual, bs = "re", k = 26), AR.start = starting_timepoint, rho = rho_value, method = "fREML", family = Gamma(link = "log").

**Model 3.3**

Accel ~ depth + s(longitude, latitude, k = 100) + s(day, k = 40) + s(time, k = 4) + s(individual, bs = "re", k = 26), AR.start = starting_point, rho = rho_value, method = "fREML", family = Gamma(link = "log").

**Model 3.4**

Accel ~ depth + s(AU1, k = 4) + s(longitude, latitude, k = 100) + s(day, k = 40) + s(time, k = 4) + s(individual, bs = "re", k = 26), AR.start = starting_point, rho = rho_value, method = "fREML", family = Gamma(link = "log").

## Results

### Hypothesis 1: Habitat use

**Fig. 2:**
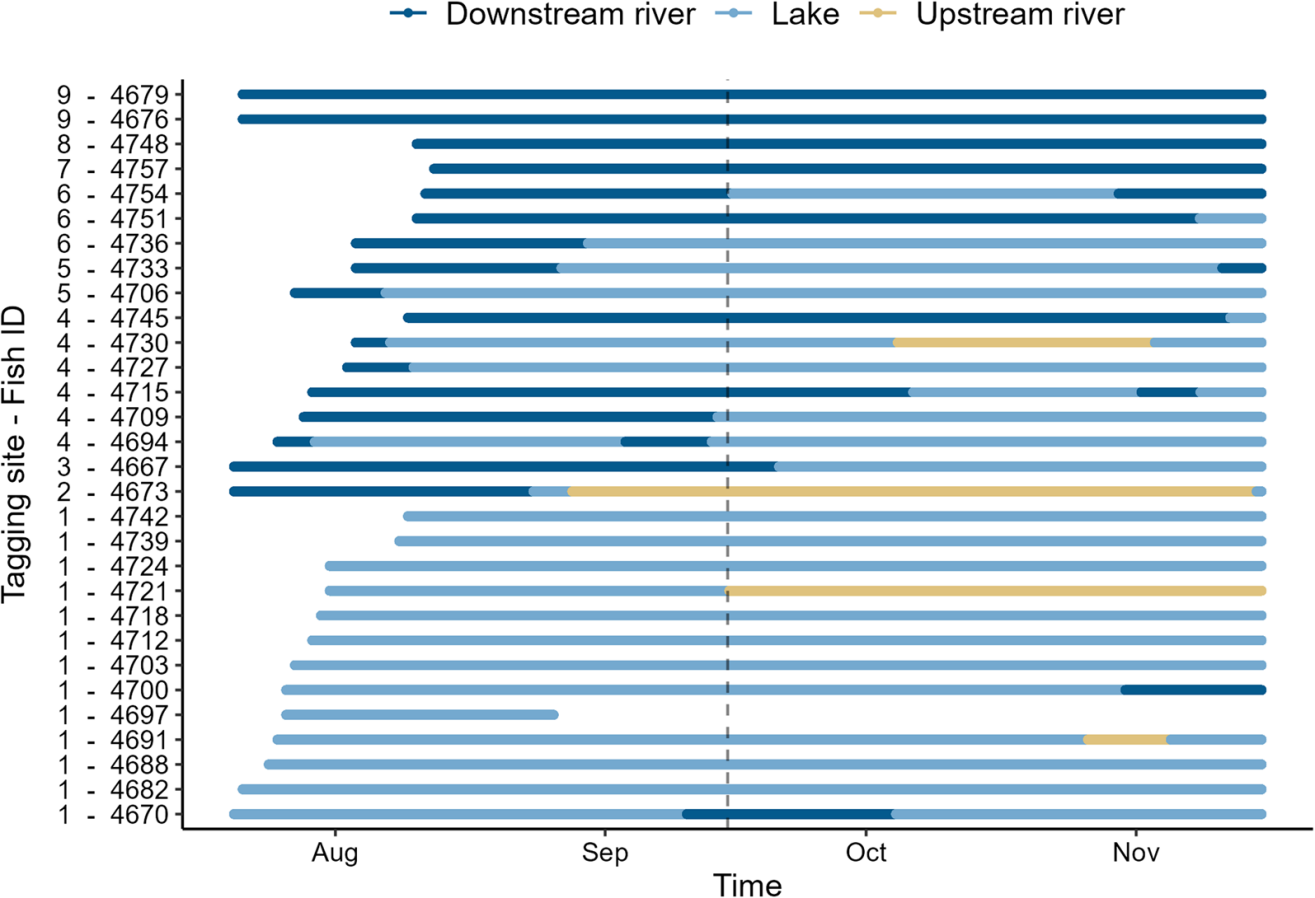
Habitat use of 30 tagged sea trout during the study period (July 20–Nov. 14, 2021, x-axis). Y-axis represents tagging site (1–9) and unique fish ID. Vertical dashed line indicates when the flap weir was elevated (Sep. 15). Tagging site 1 was at confluence of the downstream river and the lake, while tagging site 9 was close to the downstream river mouth

**Fig. 3:**
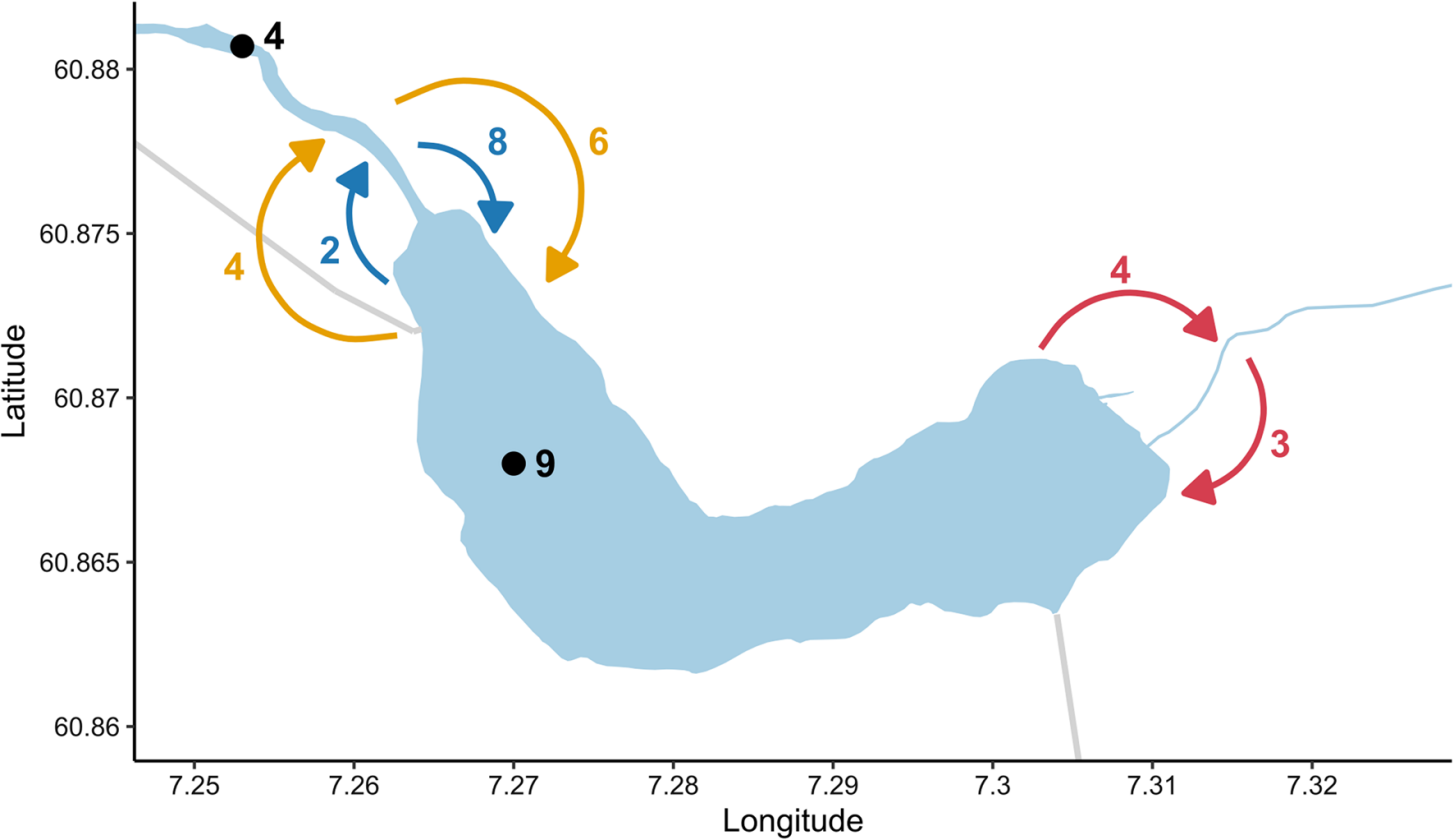
Number of sea trout moving between habitats or remained within one habitat throughout the study (July 20 to Nov. 14, 2021). Movement between downstream river and lake before (blue) and after (orange) elevation of the flap weir (Sep. 15), movement between the lake and the upstream river (red), and black points indicate how many sea trout that were only detected within the given habitat

Most of the tagged sea trout were detected in the lake (87%, N = 26), whereas nine sea trout were only detected in the lake and four sea trout only detected in the river (Figs. 2 and 3). Sea trout spent on average 83 days (SD = 34, median = 98, min = 3, max = 118) in the lake and on average 66 days (SD = 39, median = 76, min = 8, max = 118) in the rivers (Fig. 2). Among the 26 sea trout that were detected in the lake, half were tagged at the confluence of the river and the lake (N = 13) and half ascended from their tagging sites in the downstream river (N = 13, Figs. 2 and 3). The remaining 13% of the sea trout remained in the river (N = 4). A few sea trout ascended to the upstream river (13%, N = 4). Out of the sea trout tagged at the confluence, nearly 70% remained in the lake (N = 9, Figs. 2 and 3). None of the 30 sea trout were detected by the receiver at the river mouth of the downstream river (Receiver 1, Fig. 1).

Out of the sea trout that ascended to the lake, 57% ascended before (N = 8) and 43% ascended after (N = 6) the elevation of the flap weir (Sep. 15, Figs. 2 and 3). Six sea trout descended from the lake to the downstream river, in which 33% descended before (N = 2) and 77% descended after (N = 4) the elevation of the flap weir. Thus, the sea trout that ascended or descended after the flap weir was elevated used the fish ladder. All sea trout that remained in the river throughout the study ascended from their tagging site.

The generalized linear model showed that there was a significant effect of day of year on the number of sea trout in the lake (model 1.1, z = 3.031, p = 0.002), such that there were more sea trout in the lake later in the study period compared to earlier in the study period.

### Hypothesis 2: Effect of habitat on activity

**Fig. 4:**
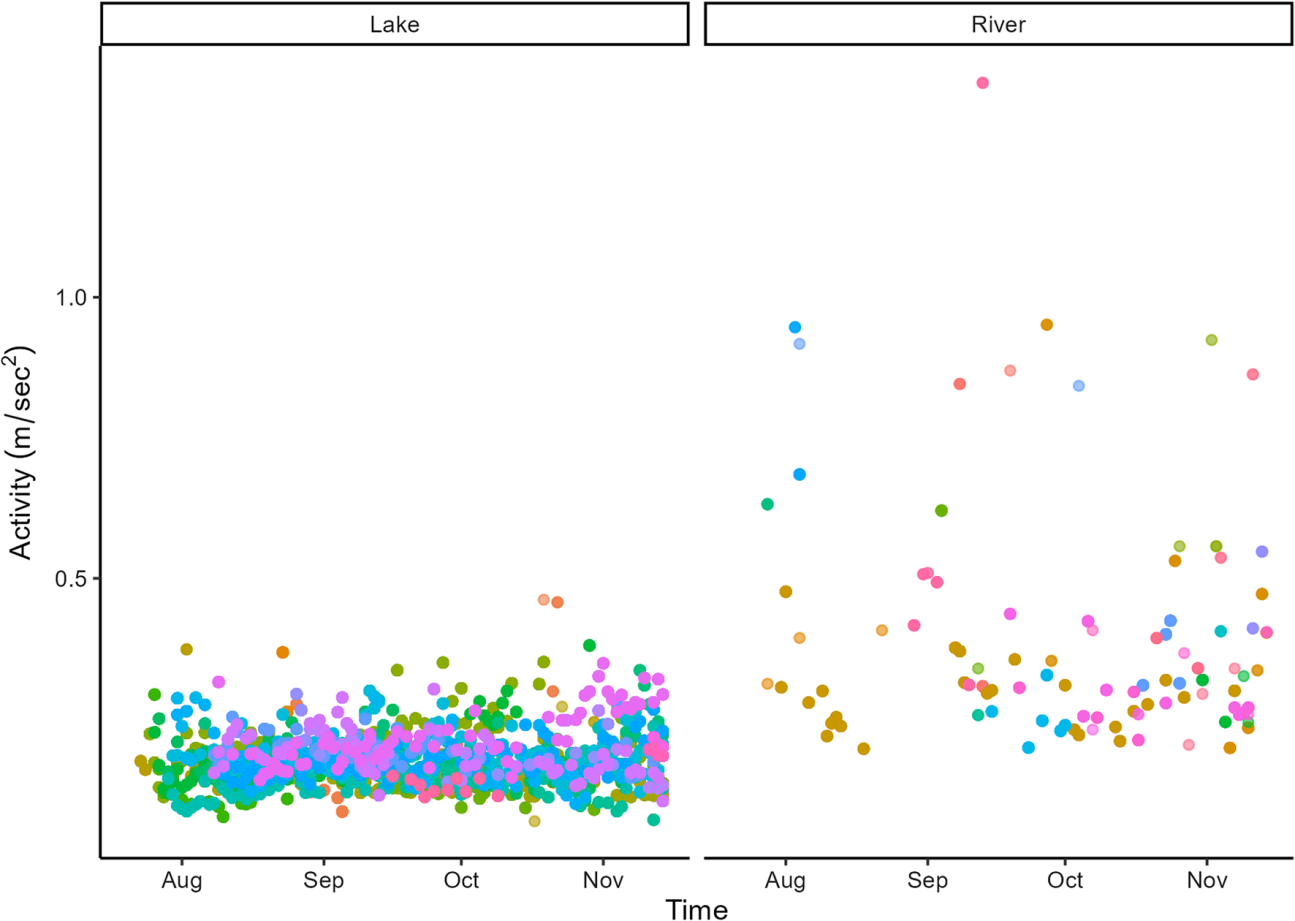
The average activity (m/s^2^) of sea trout in the two habitats: lake (Vassbygdevatnet) and river (Aurlandselva and Vassbygdelva). Colours represent different sea trout individuals

Sea trout were more active in the rivers than in the lake (Fig. 4). The predicted average activity was 0.373 m/s^2^ (SD = 0.049, median = 0.373) in the rivers and 0.183 m/s^2^ (SD = 0.016, median = 0.185) in the lake. The AIC test resulted in a lower AIC value (ΔAIC = 7014) for the model with the autocorrelation term (Model 2.2) compared to the model without (Model 2.1). Sea trout were more active during the day than during the night in both the lake and the rivers, however the effect size was small with a difference of 0.045 m/s^2^ between the least (hour = 4) and most (hour = 14) active hour of the day in the lake and a difference of 0.134 m/s^2^ between the least (hour = 4) and most (hour = 14) active hour of the day in the rivers. There was an overall decrease in the sea trout activity in the rivers throughout the study period, while the activity of sea trout in the lake slightly increased towards mid-November when data were recovered.

### Hypothesis 3: Effect of high-head storage plant discharge on behaviour in the lake

**Fig. 5:**
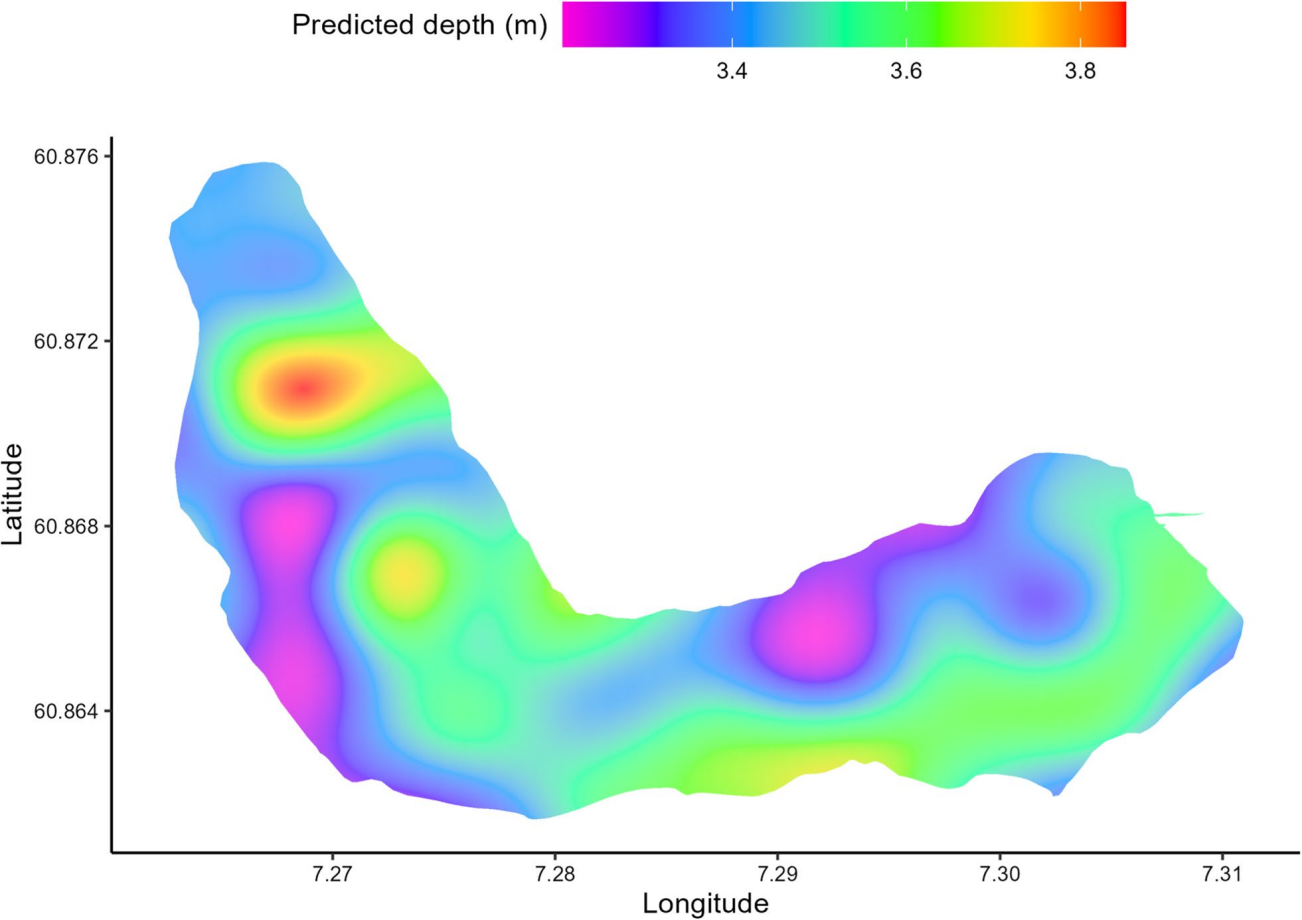
Predicted spatial depth use from the generalized additive model on the effect of discharge on the depth use of sea trout in the lake (log transformed, colour coded). Longitude and latitude on x-axis and y-axis, respectively. Warmer colour indicates deeper predicted depth use in an area

All 26 sea trout in the lake mainly utilised the upper water column throughout the study period with an overall mean depth use of 3.7 m (SD = 3.7, median = 2.6). The predicted spatial interaction revealed that sea trout showed an overall uniform shallow depth use in the lake (Fig. 5). However, most (81%) of the sea trout were at some point detected at the tag depth limit (25.5 m) during the study period. Fish length did not affect depth use in the lake. The first model that did not include the high-head storage plant discharge (Model 3.1) had a lower AIC (ΔAIC = 5895) than the model that included the discharge (Model 3.1), suggesting that the addition of discharge did not improve the model. There was an effect of individual variation in depth use. Six sea trout exploited deeper parts of the lake to a larger extent than the remaining sea trout. There was a marginal effect of time of day on depth use, such that sea trout were at deeper depths at night. The deeper habitats were used more frequently by sea trout as the study period progressed.

**Fig. 6:**
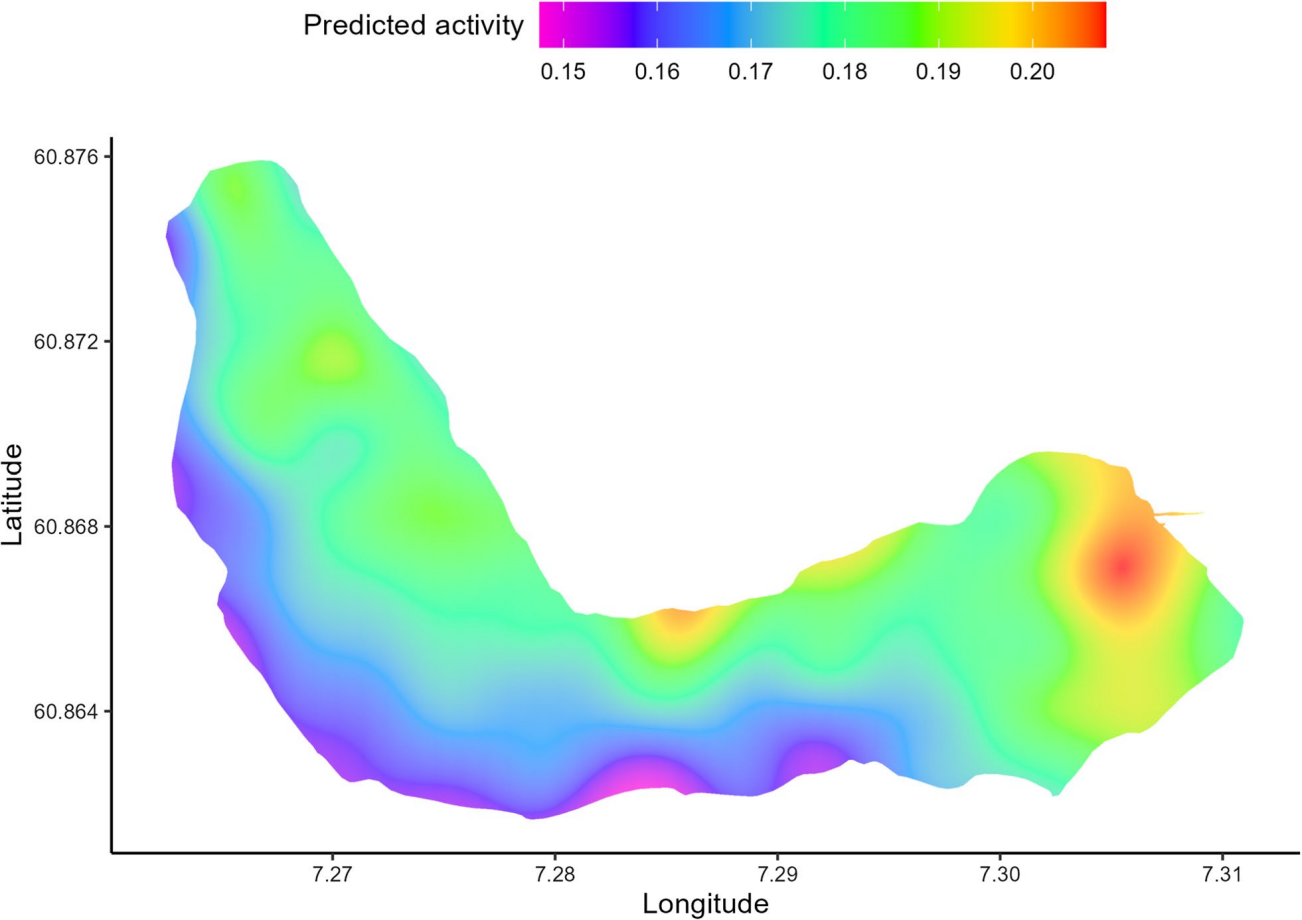
Predicted spatial activity from the generalized additive model on the effect of discharge on the activity of sea trout in the lake (log transformed, colour coded). Longitude and latitude on x-axis and y-axis, respectively. Warmer colour indicates higher activity in an area

For the models on the effect of the storage discharge on the activity of sea trout in the lake, the model including storage discharge (Model 3.4) had a better fit (ΔAIC = 5456) than the model without the discharge (Model 3.3). The discharge, however, had a minimal effect on the activity in the lake (F = 1.689, p = 0.19). There was a small increase in activity throughout the study period. Time of day had a relatively small effect on the activity, nevertheless, sea trout were more active during the day than during the night. The sea trout activity was negatively correlated with depth used such that they were less active deeper in the lake. The predicted spatial interaction on the activity of sea trout in the lake indicated that sea trout were less active around the south and southwestern areas of the lake and more active in the northern and eastern part of the lake (Fig. 6). The highest activity was in the eastern basin of the lake, where the outlet of the upstream river Vassbygdelva is located. However, there was an overall low activity level throughout the lake.

## Discussion

Vassbygdevatnet provided an important habitat for the sea trout before spawning, supporting previous findings from this lake [48]. The activity and depth of sea trout were not affected by discharge from the high-head storage plant. Ultimately, the results suggest a minimal effect from the hydropower discharge on sea trout during the period of study. Given that most sea trout inhabited the lake during the spawning migration, including the period of annual stock assessments by drift counting, prolonged residence within the lake might conceal a significant part of the sea trout population and cause an underestimation of the spawning stock biomass.

### Hypothesis 1: Habitat use

Most sea trout spent several days in the lake Vassbygdevatnet during the study, suggesting that the lake was used not only as a transition path to the upstream river, but provided an important habitat for the adult sea trout during the spawning migration. The mechanisms underlying this use, however, were not clearly revealed from this study. In rivers, pools are premium habitats used by migrating salmonids as refuge from temperature (Frechette et al. 2018) and to minimise energy expenditure [24], or as potential refuge from predators, such as European Otters (*Lutra lutra*) that preferably hunt at more narrow and shallow sites [19, 75]. As an alternative to pools, lakes such as Vassbygdevatnet can provide refuge habitat suitable for a large fraction of the population. The large number of sea trout inhabiting the lake indicates that there is an advantage to seeking refuge in the lake during the spawning migration compared to remaining in the rivers. In theory, changes to the river flow regime can affect the behaviour and distribution of fish in the watercourse, and reduce the availability of prey and spawning habitats in the rivers (as in [6, 59, 60, 82, 86]). The water level in the upstream river, Vassbygdelva, is unnaturally low due to the hydropower regulations. When the flap weir is elevated at the outlet of the lake (mid September to end of April), the discharge in Aurlandselva is artificially low and nearly constant (min flow 3 m^3^/s, [80]). Sea trout may therefore be more vulnerable to predation in the river during this low flow period (e.g., by otters; [81]). During summer, the hydropower regulations have also caused a warming in the upstream river, Vassbygdelva, coincident with a cooling in the downstream river, Aurlandselva because of water abstraction and redistribution in the system [67, 80]. Furthermore, both the downstream and upstream rivers are subject to angling during summer. Consequently, the lake may be used as a refuge by sea trout because of these anthropogenic stressors or to avoid predators. An alternative explanation is that lakes provide feeding grounds, which is observed among pre-spawning trout in Norwegian lakes [2, 27, 38, 45]. Hanssen et al. [27] documented predation of Atlantic salmon smolts by adult sea trout in lake Evangervatnet after spawning (April-June). Additionally, the lake might offer refuge for energy conservation or thermoregulation (i.e., seek certain water temperatures) before spawning [49, 51, 53]. Comparative studies between systems with and without lakes, as well as experimental manipulations of fish (e.g., displacement into or out of lakes) may help reveal the nature of the mechanistic relationships between trout and these habitats.

Although brown trout exhibit a variety of life history strategies (e.g., sea-run trout, freshwater residents; [43]), the high prevalence of sea-run trout (i.e., sea trout) in the lake in the present study is consistent with previous studies (e.g., [4, 40, 45]). In contrast, Atlantic salmon spend less time in lakes than sea trout [42, 54], despite being closely related. For instance, Atlantic salmon in the Vosso river system mainly use the lakes as aid in migration [54], while trout are abundant in the lake Evangervatnet during springtime and feeding on salmon smolts [27, 30]. Because sea trout are morphologically less adapted to strong water currents in rivers compared to Atlantic salmon [41], these two species might use freshwater habitats differently and the presence of lakes may therefore alter the competitive landscape for the two sympatric congeners. When sea trout and Atlantic salmon sympatrically inhabit river systems with lakes, competition for resources and habitat might have caused a spatial segregation whereby adult Atlantic salmon dominate in rivers and sea trout dominate in lakes. Both sea trout and Atlantic salmon inhabit the Aurland watercourse, however, the abundance of spawners differs substantially between the two species. In 2018, approximately 60 Atlantic salmon spawners and 840 sea trout spawners were registered by drift diving in the two rivers in Aurland [72, 73]. Atlantic salmon roe is stocked by a hatchery in both the upstream and downstream rivers [80], however the low abundance of Atlantic salmon spawners indicates a high mortality of Atlantic salmon at sea. The last stocking of sea trout by a hatchery was conducted in 1999 in the Aurland watercourse [80]. Thus, the lake may contribute to a better adaptation of sea trout in the watercourse in Aurland compared to Atlantic salmon.

### Hypothesis 2: Effect of habitat on activity

Sea trout were more active in the rivers than in the lake. The lower activity of sea trout in the lake indicates that the sea trout spent less energy in the lake than in the rivers (e.g., [16, 20, 47]). The high survival rate of sea trout following spawning ([9, 28] indicates that sea trout exhibit a sufficient strategy for conserving and allocating their energy. Strategic allocation and conservation of energy might be promoted by habitat preference whereby they can limit behaviours that are energy-depleting. Because the activity registered in the rivers in this study is likely caused by the active movement required to ascend rivers or maintain position against flowing water [34], sea trout likely exploited the lake Vassbygdevatnet as a habitat for energetic refuge [49, 53]. Energy expenditure during migration ultimately reduces the energy that can be used for reproduction [24].

There was temporal variation in the activity of sea trout in both lake- and river habitats. Sea trout exhibited an increase in activity throughout the study period in the lake that could be explained by spawning activity near the end. Because the spawning period of sea trout in Aurland lasts from October to early January (U. Pulg, unpublished data), the higher activity of sea trout near the end of the observation period could indicate spawning or spawning-related behaviour. Sea trout have been observed spawning in lake Vassbygdevatnet in Aurland (U. Pulg, unpublished data), and there are an increasing number of studies that document spawning in lakes in sea trout populations [12], such as in lake Røldalsvatnet, Norway [14]. Thus, the seasonal increase in activity exhibited by sea trout in the present study may potentially represent spawning or spawning-related activity in the lake.

In contrast to the observed increasing activity in the lake, there was a reduction in activity in the rivers throughout the study period. After the flap weir at the confluence of the lake and the river was elevated (Sep. 15), the water flow in the river was greatly reduced. Reduced water flow can result in a greater difficulty to migrate in rivers [79]. Berg and Berg [10] found that larger-sized sea trout resided longer at sea when the water level fell in August, which could indicate difficulty to migrate upriver. Alternatively, adult sea trout commonly seek deep pools in rivers [5, 18], where there is a lower necessity to be active due to reduced water flow. The flap weir itself also represents a migration barrier. The fish ladder is passable for fish, however bypass-fishways may restrict fish migration because they are not always easy to find [25]. Moreover, behavioural patterns, such as aggressive males at spawning sites at the fishway’s entrance may restrict fish migration. Hence, the hydropower regulations may partially explain the reduced activity of sea trout in the river.

The diel activity of sea trout was similar in the lake and the rivers. Sea trout were consistently more active during the day than at night in both habitats. Other studies have mostly found nocturnal or crepuscular peaks in activity of sea trout [13, 15, 18, 55, 93]; Barry et al. 2020), which is consistent with the diel activity of other salmonids (e.g., [29, 33, 35]). Fish are thought to be least active during the day to minimise the risk of predation by otters, birds, or piscivorous fish species. Hence, the higher activity observed during midday in both the lake and river habitats in this study contradicts theory. A higher activity of sea trout during the day in lake Vassbygdevatnet may indicate a low predation pressure on the relatively large sea trout. Alternatively, the higher activity during the day than during the night might be due to spawning or spawning-related movement (e.g., searching for spawning grounds), as have been demonstrated with Chinook salmon [50].

Receivers have a varied range that is affected by environment and climatic conditions, and swift currents likely reduce the range due to more background noise compared to calmer water areas. River receivers were therefore placed in relatively calm areas. The implication is that there is a potential bias in which we miss detections from areas where the sea trout is active, such as spawning grounds. Nevertheless, migrating fish spend most of their time holding and not actively navigating rapids or cascades that are energetically challenging [95]. However, the array still functioned well for providing a comparison between the rivers and lake as holding areas as the sea trout staged in areas for weeks or months ahead of spawning.

### Hypothesis 3: Effect of high-head storage plant discharge on behaviour in the lake

Sea trout were mostly found near the surface of the lake but showed individual variation in depth use. The varying vertical habitat used among sea trout (i.e., random effect intercept) was larger than the effect of the other parameters and contributed to explaining a large part of the variation in the data. Six sea trout used deeper depths than the remaining sea trout throughout the study. The individual variation in depth use is potentially a result of differences in personalities among sea trout. For instance, the ‘shy-bold continuum’ proposed by Wilson et al. [89] suggests that personality traits affect the observed behavioural variations among individuals. For the vertical behaviour of sea trout in lake Vassbygdevatnet, the ‘shy-bold continuum’ can potentially contribute to explaining the individual variation in depth preference. Shy individuals, compared to bold individuals, are more likely to remain at deeper depths to limit their exposure to threats (e.g., fishing, terrestrial or avian predators). Additionally, the individual vertical movement differences observed in the present study might be a result of individual fitness because vertical movement is costly [78] or a result of food availability.

The high-head storage plant discharge did not influence depth use of sea trout in the lake. There were, however, temporal effects on the depth use of sea trout in the lake, such that sea trout displayed greater depth use as the study progressed. In comparison, the diel temporal effect on the depth use in the lake was smaller. Sea trout were detected more frequently at shallow water depth during the day than during the night, which is aligned with the activity peak of the sea trout in the present study. Because sea trout are visual feeders [43], sea trout might utilise daylight to feed at the surface.

The high-head storage plant discharge did not affect the activity of sea trout in the lake. Because the inflow of water from the high-head storage plant affects the stratification of the lake [80], and temperature is closely related to energy consumption and activity [17], it is likely that there is an effect of discharge on the activity of sea trout that is not accounted for by the change in discharge. Although the addition of discharge improved the model on the activity of sea trout in the lake, the effect was small, and the shape of the fit was seemingly impacted by a few extreme values of discharge that were rarely encountered by the sea trout during the study period. Thus, the discharge from the high-head storage plant had no evident effect on the activity of sea trout in the lake.

The higher activity of sea trout observed in the eastern part of the lake may indicate that there was an effect from the high-head storage plant discharge, despite the model not accounting for the discharge location directly. The additional supply of water from the high-head storage plant into the surface layer of the lake caused a higher surface flow that could result in an increase in sea trout activity, particularly around the discharge area. Swimming towards discharging water will require higher activity, similar to the demands of holding position against the flow in a river. Thus, it is likely that the observed increase in activity around the Aurland 1 discharge is related to the outflow of water.

### Implications for management

The large prevalence of sea trout inhabiting the lake during the spawning migration demonstrates that the lake provided an important habitat for sea trout, where they likely conserved energy and found refuge from predators prior to spawning. Based on factors, such as hydropower regulations, overfishing, and sea lice from open net pen aquaculture in the fjords, assessment of Norwegian sea trout populations has concluded that only 25% of the populations are in a good condition [83, 84]. The sea trout assessment is based on drift diving in rivers [74]. Given that most sea trout inhabited the lake during the spawning migration, including the period of annual stock assessments, the lake might conceal a significant part of the sea trout population and cause an underestimation of the spawning stock biomass. Thus, lake-residing fish should be taken into consideration when management efforts are made based on spawning stocks. For example, stock assessment of several Norwegian river systems might be underestimated given that about 30% of river systems in Norway contain lakes [27].

With the increasing demand of renewable energy, lakes are likely to become increasingly exploited as reservoirs for hydropower [32]. Given that lakes provide such important habitat for sea trout, effects of hydropower on this habitat may render sea trout particularly vulnerable. However, the effect of hydropower regulations on the lake ecology of salmonids is poorly documented [46], despite being among the most frequently studied fish species globally [12]. Because sea trout and Atlantic salmon exhibit different life history strategies [43], hydropower mitigation efforts based on the ecology of Atlantic salmon can misrepresent the requirements of sea trout. Consequently, current management mitigations and regulations might not be sufficient if they fail to consider the unique ecology of trout. Thus, management and the hydropower industry should further invest in research on the lake ecology of sea trout to provide necessary knowledge on the requirements of sea trout populations.

## Conclusion

This study demonstrated that the lake offered an important habitat for sea trout during their spawning migration based on acoustic detections and position calculations with YAPS. The activity of sea trout was higher in the rivers than in the lake, indicating that the lake offered a refuge where sea trout could conserve energy during the holding phase of migration as the fish prepared for spawning. Additionally, there was a seasonal difference in activity of sea trout between the lake and river habitats; the activity of sea trout peaked earlier in the rivers than in the lake. This could indicate that spawning or spawning-related movement might have occurred in the lake as the spawning period approached. There was not an effect of discharge from the high-head storage plant on depth use or activity of sea trout in the lake. Our results indicate that trout have low activity in the lake compared to the river and may use lake habitats as a refuge during their stay in freshwater, which may have carryover benefits to the animals that use the lake, which have not yet been revealed from this research. In a regulated river where the hydrodynamic condition is altered, one could expect trout to use the lake more. Although we have revealed little direct impact of the discharge from the hydropower plant, further research on the effect of storage plants and its facilities on the lake and migration behaviour of fish is needed.

### Supplementary Information

**Additional file 1: Figure S1**. Average discharge data from the high-head storage plant ‘Aurland 1’ during the study period, July 20. to Nov. 14., 2021. Average daily discharge data (dark blue) and overall average discharge during the study period (light blue straight line). Time on x-axis and water discharge (m^3^/s) on y-axis.

## Data Availability

All telemetry data are available through the Ocean Tracking Network.

## References

[1] Alfredsen K, Amundsen PA, Hahn L (2022). A synoptic history of the development, production and environmental oversight of hydropower in Brazil, Canada, and Norway. Hydrobiologia.

[2] Amundsen PA, Knudsen R (2009). Winter ecology of Arctic charr (Salvelinus alpinus) and brown trout (Salmo trutta) in a subarctic lake, Norway. Aquat Ecol.

[3] Anderson D, Moggridge H, Warren P, Shucksmith J (2015). The impacts of ‘run-of-river’ hydropower on the physical and ecological condition of rivers. Water Environ J.

[4] Andersson A, Greenberg LA, Bergman E, Su Z, Andersson M, Piccolo JJ. Recreational trolling effort and catch of Atlantic salmon and brown trout in Vänern, the EU’s largest lake. In Fisheries Research 2020;(Vol. 227, p. 105548). Elsevier BV. 10.1016/j.fishres.2020.105548

[5] Arnekleiv JV, Rønning L (2004). Migratory patterns and return to the catch site of adult brown trout (Salmo trutta L.) in a regulated river. River Res Appl.

[6] Banks JW (1969). A review of the literature on the upstream migration of adult salmonids. J Fish Biol.

[7] Baktoft H, Gjelland KØ, Økland F, Thygesen UH (2017). Positioning of aquatic animals based on time-of-arrival and random walk models using YAPS (Yet Another Positioning Solver). Sci Rep.

[8] Belletti B, Garcia de Leaniz C, Jones J, Bizzi S, Börger L, Segura G, Castelletti A, van de Bund W, Aarestrup K, Barry J, Belka K, Berkhuysen A, Birnie-Gauvin K, Bussettini M, Carolli M, Consuegra S, Dopico E, Feierfeil T, Fernández S, Giannico G. More than one million barriers fragment Europe's rivers. Nature (London) 2020;588(7838), 436–441. 10.1038/s41586-020-3005-2 10.1038/s41586-020-3005-2 33328667

[9] Bendall B, Moor A, Quayle V (2005). The post-spawning movements of migratory brown trout Salmo trutta L. J Fish Biol..

[10] Berg OK, Berg M (1989). Sea growth and time of migration of anadromous Arctic char (Salvelinus alpinus) from the Vardnes River, in northern Norway. Can J Fish Aquat Sci.

[11] Birnie-Gauvin K, Candee M, Baktoft H, Larsen M, Koed A, Aarestrup K (2018). River connectivity reestablished: Effects and implications of six weir removals on brown trout smolt migration. River Res Appl.

[12] Birnie-Gauvin K, Thorstad EB, Aarestrup K (2019). Overlooked aspects of the Salmo salar and Salmo trutta lifecycles. Rev Fish Biol Fish.

[13] Björnsson B (2001). Diel changes in the feeding behaviour of Arctic char (Salvelinus alpinus) and brown trout (Salmo trutta) in Ellidavatn, a small lake in southwest Iceland. Limnologica.

[14] Brabrand Å, Koestler AG, Borgstrøm R (2002). Lake spawning of brown trout related to groundwater influx. J Fish Biol.

[15] Bremset G (2000). Seasonal and Diel Changes in Behaviour, Microhabitat use and Preferences by Young Pool-dwelling Atlantic Salmon, Salmo salar, and Brown Trout, Salmo Trutta. Environ Biol Fishes.

[16] Briggs CT, Post JR (1997). In situ activity metabolism of rainbow trout (Oncorhynchus mykiss): estimates obtained from telemetry of axial muscle electromyograms. Can J Fish Aquat Sci.

[17] Brown JH, Gillooly JF, Allen AP, Savage VM, West GB (2004). Toward a metabolic theory of ecology. Ecology.

[18] Bunnell DB, Isely JJ, Burrell KH, Van Lear DH (1998). Diel Movement of Brown Trout in a Southern Appalachian River. Trans Am Fish Soc.

[19] Cho HS, Choi KH, Lee SD, Park YS (2009). Characterizing habitat preference of Eurasian river otter (Lutra lutra) in streams using a self-organizing map. Limnology.

[20] Cooke SJ, Brownscombe JW, Raby GD, Broell F, Hinch SG, Clark TD, Semmens JM (2016). Remote bioenergetics measurements in wild fish: opportunities and challenges. Comp Biochem Physiol Part A Mol Integr Physiol.

[21] Cuthbert RN, Diagne C, Hudgins EJ, Turbelin A, Ahmed DA, Albert C, Bodey TW, Briski E, Essl F, Haubrock PJ, Gozlan RE, Kirichenko N, Kourantidou M, Kramer AM, Courchamp F (2022). Biological invasion costs reveal insufficient proactive management worldwide. Sci Total Environ.

[22] Dudgeon D, Arthington AH, Gessner MO, Kawabata Z-I, Knowler DJ, Lévêque C, Naiman RJ, Prieur-Richard A-H, Soto D, Stiassny MLJ, Sullivan CA (2006). Freshwater biodiversity: importance, threats, status and conservation challenges. Biol Rev Camb Philos Soc.

[23] Farrell AP, Lee CG, Tierney K, Hodaly A, Clutterham S, Healey M, Hinch S, Lotto A (2003). Field-based measurements of oxygen uptake and swimming performance with adult Pacific salmon using a mobile respirometer swim tunnel. J Fish Biol.

[24] Fenkes M, Shiels HA, Fitzpatrick JL, Nudds RL (2016). The potential impacts of migratory difficulty, including warmer waters and altered flow conditions, on the reproductive success of salmonid fishes. Comp Biochem Physiol A Mol Integr Physiol.

[25] Fjeldstad HP, Pulg U, Forseth T (2018). Safe two-way migration for salmonids and eel past hydropower structures in Europe: a review and recommendations for best-practice solutions. Marine Freshwater Res..

[26] Grill G, Lehner B, Thieme M, Geenen B, Tickner D, Antonelli F, Babu S, Borrelli P, Cheng L, Crochetiere H, Ehalt Macedo H, Filgueiras R, Goichot M, Higgins J, Hogan Z, Lip B, McClain ME, Meng J, Mulligan M, Zarfl C (2019). Mapping the world's free-flowing rivers. Nature.

[27] Hanssen EM, Vollset KW, Salvanes AGV, Barlaup B, Whoriskey K, Isaksen TE, Normann ES, Hulbak M, Lennox RJ (2022). Acoustic telemetry predation sensors reveal the tribulations of Atlantic salmon ( Salmo salar ) smolts migrating through lakes. Ecol Freshw Fish.

[28] Haraldstad T, Höglund E, Kroglund F, Lamberg A, Olsen EM, Haugen TO (2018). Condition-dependent skipped spawning in anadromous brown trout (Salmo trutta). Can J Fish Aquat Sci.

[29] Harrison PM, Gutowsky LFG, Martins EG, Patterson DA, Leake A, Cooke SJ, Power M (2013). Diel vertical migration of adult burbot: a dynamic trade-off among feeding opportunity, predation avoidance, and bioenergetic gain. Can J Fish Aquat Sci.

[30] Haugen TO, Kristensen T, Nilsen TO, Urke HA (2017). Vandringsmønsteret til laksesmolt i Vossovassdraget med vekt på detaljert kartlegging av åtferd i innsjøsystema og effektar av miljøtilhøve. MINA Fagrapport.

[31] Heggenes J, Stickler M, Alfredsen K, Brittain JE, Adeva-Bustos A, Huusko A (2021). Hydropower-driven thermal changes, biological responses and mitigating measures in northern river systems. River Res Appl.

[32] Hirsch PE, Eloranta AP, Amundsen PA (2017). Effects of water level regulation in alpine hydropower reservoirs: an ecosystem perspective with a special emphasis on fish. Hydrobiologia.

[33] Huusko A, Greenberg L, Stickler M, Linnansaari T, Nykänen M, Vehanen T, Koljonen S, Louhi P, Alfredsen K (2007). Life in the ice lane: the winter ecology of stream salmonids. River Res Appl.

[34] Hynes HBN (1970). The ecology of running waters.

[35] Jakober MJ, McMahon TE, Thurow RF (2000). Diel habitat partitioning by bull charr and cutthroat trout during fall and winter in rocky mountain streams. Environ Biol Fishes.

[36] Jeffrey JD, Hasler CT, Chapman JM, Cooke SJ, Suski CD. Linking landscape-scale disturbances to stress and condition of fish: implications for restoration and conservation. In Integrative and Comparative Biology 2015; (Vol. 55, Issue 4, pp. 618–630). Oxford University Press (OUP). 10.1093/icb/icv022 10.1093/icb/icv022 25931612

[37] Jensen AJ, Johnsen BO, Møkkelgjerd PI (1993). Sjøaure og laks i Aurlandsvassdraget 1911–92. NINA Forskningsrapport.

[38] Jensen H, Kiljunen M, Amundsen P-A (2012). Dietary ontogeny and niche shift to piscivory in lacustrine brown trout Salmo trutta revealed by stomach content and stable isotope analyses. J Fish Biol.

[39] Johnson JB, Omland KS (2004). Model selection in ecology and evolution. Trends Ecol Evol.

[40] Jonsson B (1989). Life history and habitat use of Norwegian brown trout (Salmo trutta). Freshw Biol.

[41] Jonsson B, Jonsson N (2011). Ecology of Atlantic Salmon and Brown Trout. Springer, Netherlands.

[42] Kennedy R, Allen M (2016). The pre-spawning migratory behaviour of Atlantic salmon Salmo salar in a large lacustrine catchment. J Fish Biol.

[43] Klemetsen A, Amundsen P-A, Dempson JB, Jonsson B, Jonsson N, O’Connell MF, Mortensen E (2003). Atlantic salmon Salmo salar L., brown trout Salmo trutta L. and Arctic charr Salvelinus alpinus (L.): a review of aspects of their life histories. Ecol Freshw Fish.

[44] Koed A, Birnie-Gauvin K, Sivebæk F, Aarestrup K (2019). From endangered to sustainable: multi-faceted management in rivers and coasts improves Atlantic salmon (Salmo salar) populations in Denmark. Fish Manag Ecol.

[45] L'Abée-Lund JH, Langeland A, Sægrov H (1992). Piscivory by brown trout Salmo trutta L. and Arctic charr Salvelinus alpinus (L.) in Norwegian lakes. J Fish Biol.

[46] Lennox RJ, Pulg U, Malley B, Gabrielsen SE, Hanssen SM, Cooke SJ, Birnie-Gauvin K, Barlaup BT, Vollset KW (2021). The various ways that anadromous salmonids use lake habitats to complete their life history. Can J Fish Aquat Sci.

[47] Lowe CG, Holland KN, Wolcott TG (1998). A new acoustic tailbeat transmitter for fishes. Fish Res.

[48] Lunde R. Lake-habitat use of post-juvenile sea trout over time and space—An acoustic telemetry study in a regulated river. Master’s thesis. Norwegian University of Life Sciences, Ås;2014.

[49] Mathes MT, Hinch SG, Cooke SJ, Crossin GT, Patterson DA, Lotto AG, Farrell AP (2010). Effect of water temperature, timing, physiological condition, and lake thermal refugia on migrating adult Weaver Creek sockeye salmon (Oncorhynchus nerka). Can J Fish Aquat Sci.

[50] McMichael GA, McKinstry CA, Vucelick JA, Lukas JA (2005). Fall chinook salmon spawning activity versus daylight and flow in the tailrace of a large hydroelectric dam. N Am J Fish Manag.

[51] Mulder IM, Morris CJ, Dempson JB, Fleming IA, Power M. Overwinter thermal habitat use in lakes by anadromous Arctic char. In Canadian Journal of Fisheries and Aquatic Sciences 2018; (Vol. 75, Issue 12, pp. 2343–2353). Canadian Science Publishing. 10.1139/cjfas-2017-0420

[52] Mulder IM, Dempson JB, Fleming IA, Power M (2019). Diel activity patterns in overwintering Labrador anadromous Arctic charr. Hydrobiologia.

[53] Newell JC, Quinn TP (2005). Behavioral thermoregulation by maturing adult sockeye salmon (Oncorhynchus nerka) in a stratified lake prior to spawning. Can J Zool.

[54] Nilsen CI, Vollset KW, Velle G, Barlaup BT, Normann ES, Stöger E, Lennox RJ (2022). Atlantic salmon of wild and hatchery origin have different migration patterns. Can J Fish Aquat Sci.

[55] Ovidio M, Baras E, Goffaux D, Giroux F, Philippart JC (2002). Seasonal variations of activity pattern of brown trout (Salmo trutta) in a small stream, as determined by radio-telemetry. Hydrobiologia.

[56] Palstra A, Kals J, Böhm T, Bastiaansen JW, Komen H (2020). Swimming performance and oxygen consumption as non-lethal indicators of production Traits in Atlantic Salmon and Gilthead Seabream. Front Physiol.

[57] Pedersen EJ, Miller DL, Simpson GL, Ross N (2019). Hierarchical generalized additive models in ecology: an introduction with mgcv. PeerJ..

[58] Peiman KS, Birnie-Gauvin K, Midwood JD, Larsen MH, Wilson ADM, Aarestrup K, Cooke SJ (2017). If and when: intrinsic differences and environmental stressors influence migration in brown trout (Salmo trutta). Oecologia.

[59] Poff NL, Allan JD, Bain MB, Karr JR, Prestegaard KL, Richter BD, Sparks RE, Stromberg JC (1997). The natural flow regime. Bioscience.

[60] Poff NL, Hart DD (2002). How dams vary and why it matters for the emerging science of dam removal. Bioscience.

[61] Pulg U, Barlaup BT, Sternecker K, Trepl L, Unfer G (2011). Restoration of spawning habitats of brown trout (Salmo trutta) in a regulated chalk stream. River Res Applic.

[62] Pulg U, Stranzl S, Olsen EE, Postler C. Vanndekt areal, habitatkvalitet og vannføring i Vassbygdelva (2020). NORCE LFI rapport 379. NORCE LFI, Bergen. (In Norwegian).

[63] Pulg U, Lennox RJ, Stranzl S, Espedal EO, Gabrielsen SE, Wiers T, Velle G, Hauer C, Dønnum BO, Barlaup BT (2022). Long-term effects and cost-benefit analysis of eight spawning gravel augmentations for Atlantic salmon and Brown trout in Norway. Hydrobiologia.

[64] R Core Team. R: A language and environment for statistical computing. R Foundation for Statistical Computing, Vienna, Austria;2021. https://www.R-project.org/.

[65] Reid AJ, Carlson AK, Creed IF, Eliason EJ, Gell PA, Johnson PTJ, Kidd KA, MacCormack TJ, Olden JD, Ormerod SJ, Smol JP, Taylor WW, Tockner K, Vermaire JC, Dudgeon D, Cooke SJ (2019). Emerging threats and persistent conservation challenges for freshwater biodiversity. Biol Rev.

[66] Roni P, Hanson K, Beechie T (2008). Global review of the physical and biological effectiveness of stream habitat rehabilitation techniques. N Am J Fish Manag.

[67] Saltveit SJ (2006). Økologiske forhold i vassdrag: konsekvenser av vannføringsendringer: en sammenstilling av dagens kunnskap (in Norwegian).

[68] Schloerke B, Cook D, Larmarange J, Briatte F, Marbach M, Thoen E, Elberg A, Crowley J. GGally: Extension to 'ggplot2'. R package version 2.1.2; 2021. https://CRAN.R-project.org/package=GGally

[69] Schwinn M, Aarestrup K, Baktoft H, Koed A (2017). Survival of migrating sea trout (Salmo trutta) smolts during their passage of an artificial lake in a danish lowland stream. River Res Appl.

[70] Simpfendorfer CA, Huveneers C, Steckenreuter A, Tattersall K, Hoenner X, Harcourt R, Heupel MR (2015). Ghosts in the data: false detections in VEMCO pulse position modulation acoustic telemetry monitoring equipment. Anim Biotelem.

[71] Simpson G. gratia: Graceful ggplot-Based Graphics and Other Functions for GAMs Fitted using mgcv. R package version 0.6.0; 2021. https://gavinsimpson.github.io/gratia/.

[72] Skoglund H, Vollset KW, Barlaup B, Lennox R. Gytefisktelling av laks og sjøaure på Vestlandet status og utvikling i perioden 2004–2018. NORCE LFI rapport 357. Norwegian Research Centre Laboratorium for ferskvannsøkologi og innlandsfiske; 2019a.

[73] Skoglund H, Wiers T, Normann ES, Stranzl S, Landro Y, Pulg U, Velle G, Gabrielsen SE, Lehman GB, Barlaup BT. Gytefisktelling av laks og sjøaure og uttak av rømt oppdrettslaks i 49 elver på Vestlandet høsten 2018. NORCE LFI rapport 359. Norwegian Research Centre Laboratorium for ferskvannsøkologi og innlandsfiske; 2019b.

[74] Skoglund H, Vollset KW, Lennox R, Skaala Ø, Barlaup BT (2021). Drift diving: a quick and accurate method for assessment of anadromous salmonid spawning populations. Fish Manag Ecol.

[75] Sortland LK, Lennox RJ, Velle G, Vollset KW, Kambestad M. Impacts of predation by Eurasian otters on Atlantic salmon in two Norwegian rivers. Freshw Biol 2023. 10.1111/fwb.14095

[76] Smircich MG, Kelly JT (2014). Extending the 2% rule: the effects of heavy internal tags on stress physiology, swimming performance, and growth in brook trout. Anim Biotelem.

[77] Stanford JA, Ward JV, Liss WJ, Frissell CA, Williams RN, Lichatowich JA, Coutant CC (1996). A general protocol for restoration of regulated rivers. Regul Rivers Res Mgmt.

[78] Strand E, Jørgensen C, Huse G (2005). Modelling buoyancy regulation in fishes with swimbladders: bioenergetics and behaviour. Ecol Model.

[79] Thorstad EB, Økland F, Kroglund F, Jepsen N (2003). Upstream migration of Atlantic salmon at a power station on the River Nidelva, Southern Norway. Fish Manag Ecol.

[80] Ugedal O, Pulg U, Skoglund H, Charmasson J, Espedal EO, Jensås JG, Stranzl S, Harby A, Forseth T. Sjøaure og laks i Aurlandsvassdraget 2009–2018. Reguleringseffekter, miljødesign og tiltak (No. NINA Rapport 1716). Norsk institutt for naturforskning;2019.

[81] Van Dijk J, Kambestad M, Carss DC, Hamre Ø. Kartlegging av oterens effekt på bestander av laks og sjøørret—Sunnmøre. NINA Rapport 1780. Norsk institutt for naturforskning; 2020.

[82] Vannote RL, Minshall GW, Cummins KW, Sedell JR, Cushing CE (1980). The river continuum concept. Can J Fish Aquat Sci.

[83] VRL (Vitenskapelig råd for lakseforvaltning). (2019). Klassifisering av tilstanden til 430 norske sjøørretbestander. Temarapport fra Vitenskapelig råd for lakseforvaltning nr 7, 150 s.

[84] VRL (Vitenskapelig råd for lakseforvaltning). (2022). Klassifisering av tilstanden til sjøørret i 1279 vassdrag. Temarapport fra Vitenskapelig råd for lakseforvaltning nr 9, 170 s.

[85] Werner EE, Anholt BR (1993). Ecological consequences of the trade-off between growth and mortality rates mediated by foraging activity. Am Nat.

[86] Westrelin S, Roy R, Tissot-Rey L, Bergès L, Argillier C (2017). Habitat use and preference of adult perch (*Perca fluviatilis* L.) in a deep reservoir: variations with seasons, water levels and individuals. Hydrobiologia.

[87] Wickham H (2016). ggplot2: elegant graphics for data analysis.

[88] Wickham H, François R, Henry L, Müller K. dplyr: a grammar of data manipulation. R package version 1.0.9;2022. https://CRAN.R-project.org/package=dplyr

[89] Wilson DS, Coleman K, Clark AB, Biederman L. Shy-bold continuum in pumpkinseed sunfish (Lepomis gibbosus): An ecological study of a psychological trait. In Journal of Comparative Psychology (Vol. 107, Issue 3, pp. 250–260);1993. American Psychological Association (APA). 10.1037/0735-7036.107.3.250

[90] Wood SN. Generalized Additive Models: An Introduction with R (2nd edition). Chapman and Hall/CRC;2017.

[91] WWF. Living Planet Report 2020—Bending the curve of biodiversity loss. In: Almond REA, Grooten M, Petersen T (Eds). WWF, Gland, Switzerland;2020.

[92] Zuur AF, Ino EN, Walker NJ, Saveliev AA, Smith GM (2009). Mixed effects models and extensions in ecology with R.

[93] Young MK (1999). Summer diel activity and movement of adult brown trout in high-elevation streams in Wyoming, USA. J Fish Biol.

[94] Økland F, Jensen AJ, Johnsen BO (1995). Vandring hos radiomerket ørret i Aurlandsvassdraget.- Vandrer sjøørret inn i Vangen kraftverk?. NINA Oppdragsmelding.

[95] Økland F, Erkinaro J, Moen K, Niemelä E, Fiske P, McKinley R, Thorstad EB (2001). Return migration of Atlantic Salmon in the River Tana: phases of migratory behaviour. J Fish Biol.

