# Supplementary figures and images for: Behaviour of anadromous brown trout (Salmo trutta) in a hydropower regulated freshwater system
Source: Mov Ecol. 2023 Oct 14;11:63. doi: 10.1186/s40462-023-00429-7 (PMC10576395; doi:10.1186/s40462-023-00429-7)

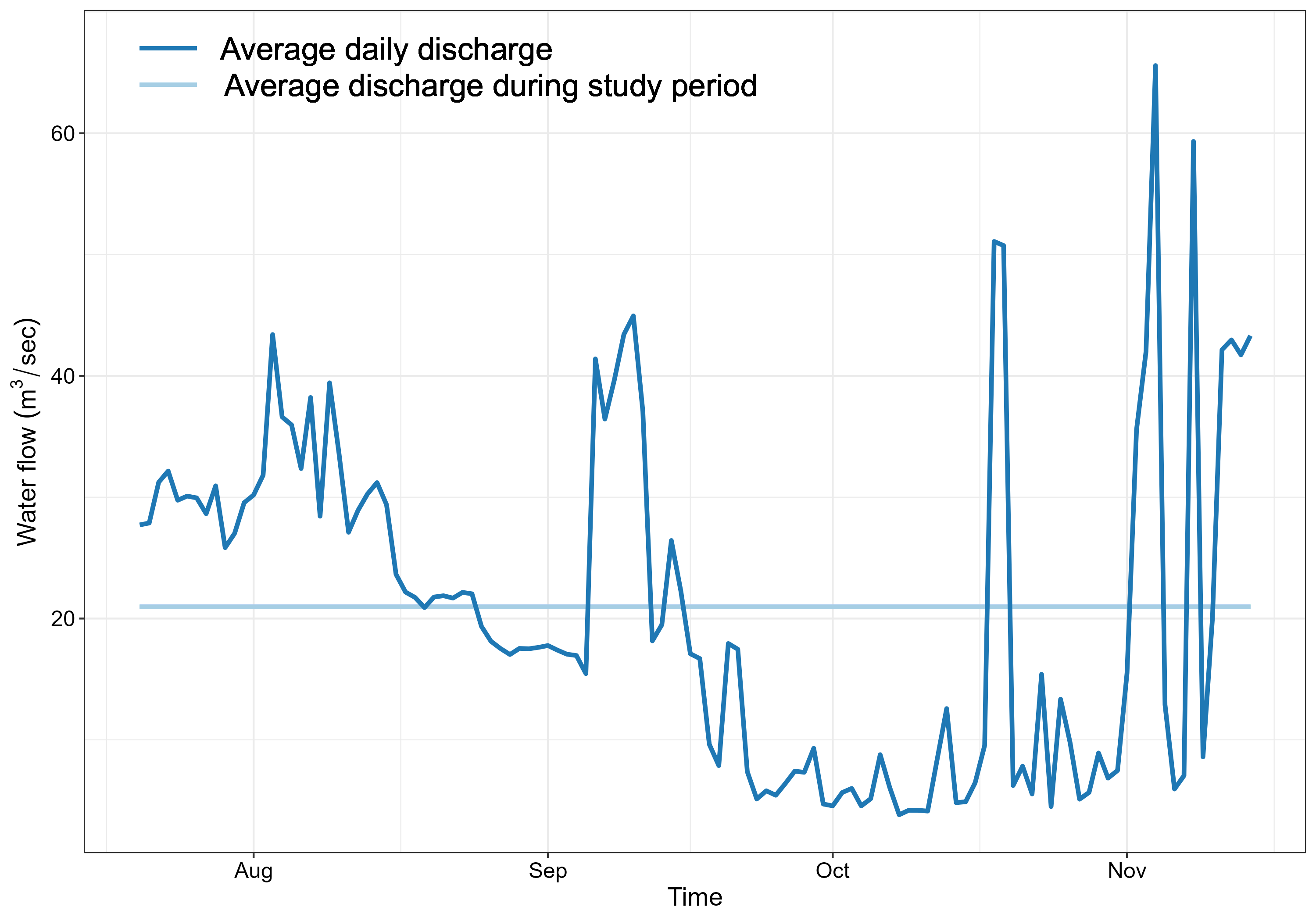

Supplement: Supplementary file 1 — Additional file 1: Figure S1. Average discharge data from the high-head storage plant ‘Aurland 1’ during the study period, July 20. to Nov. 14., 2021. Average daily discharge data (dark blue) and overall average discharge during the study period (light blue straight line). Time on x-axis and water discharge (m3/s) on y-axis. [file 40462_2023_429_MOESM1_ESM.tiff]
